# Causal relationship between systemic lupus erythematosus and primary liver cirrhosis based on two-sample bidirectional Mendelian randomization and transcriptome overlap analysis

**DOI:** 10.1186/s13075-023-03235-z

**Published:** 2024-01-02

**Authors:** Linyong Wu, Songhua Li, Chaojun Wu, Shaofeng Wu, Yan Lin, Dayou Wei

**Affiliations:** Department of Medical Ultrasound, Maoming People’s Hospital, Maoming, Guangdong 525000 People’s Republic of China

**Keywords:** Systemic lupus erythematosus, Primary biliary cirrhosis, Mendelian randomization, Transcriptome overlap analysis

## Abstract

**Background:**

Overlapping cases of systemic lupus erythematosus (SLE) and primary biliary cirrhosis (PBC) are rare and have not yet been fully proven to be accidental or have a common genetic basis.

**Methods:**

Two-sample bidirectional Mendelian randomization (MR) analysis was applied to explore the potential causal relationship between SLE and PBC. The heterogeneity and reliability of MR analysis were evaluated through Cochran’s *Q*-test and sensitivity test, respectively. Next, transcriptome overlap analysis of SLE and PBC was performed using the Gene Expression Omnibus database to identify the potential mechanism of hub genes. Finally, based on MR analysis, the potential causal relationship between hub genes and SLE or PBC was validated again.

**Results:**

The MR analysis results indicated that SLE and PBC were both high-risk factors for the occurrence and development of the other party. On the one hand, MR analysis had heterogeneity, and on the other hand, it also had robustness. Nine hub genes were identified through transcriptome overlap analysis, and machine learning algorithms were used to verify their high recognition efficiency for SLE patients. Finally, based on MR analysis, it was verified that there was no potential causal relationship between the central gene SOCS3 and SLE, but it was a high-risk factor for the potential risk of PBC.

**Conclusion:**

The two-sample bidirectional MR analysis revealed that SLE and PBC were high-risk factors for each other, indicating that they had similar genetic bases, which could to some extent overcome the limitation of insufficient overlap in case samples of SLE and PBC. The analysis of transcriptome overlapping hub genes provided a theoretical basis for the potential mechanisms and therapeutic targets of SLE with PBC overlapping cases.

## Background

Autoimmune diseases are all caused by the loss of immune tolerance, and genetic susceptibility is related to the loss of tolerance and autoimmunity. Autoimmune diseases may have similar genetic susceptibility, and multiple autoimmune diseases can occur in the same patient [1]. Systemic lupus erythematosus (SLE) and primary biliary (PBC) cirrhosis are both chronic autoimmune diseases.

SLE is an immune disease with multiple system involvement, recurrent attacks, diverse clinical manifestations, and high incidence rate in women. SLE is also often accompanied by other immune diseases. For example, the prevalence of SLE with Sjogren’s syndrome exceeded 20% based on two different assessment criteria [2]. The liver is an important target organ involved in multiple systems of SLE. Liver involvement in SLE can also lead to liver dysfunction, which can further lead to cirrhosis and liver cancer. However, the diagnosis and treatment of liver involvement in SLE may not be timely, which masks the progression of liver involvement. In addition, SLE inhibition therapy may also mask the progression of primary liver disease. There are still difficulties in accurately distinguishing SLE from primary liver diseases that are difficult to overcome.

PBC is a primary autoimmune disease of the liver that is more common in women, characterized by cholestasis, bile duct injury, cholangitis, and liver fibrosis, which can also lead to liver dysfunction, cirrhosis, or liver cancer [3]. PBC is also accompanied by extrahepatic autoimmune diseases. For example, a systematic review found that the prevalence of PBC associated with Sjogren’s syndrome ranged from 3.5 to 73%. However, the low incidence of PBC has rendered many existing treatments ineffective for PBC. If combined with extrahepatic autoimmune diseases, this will pose greater challenges for the precise treatment of PBC [4].

The overview of SLE and PBC mentioned above indicates that there are many similarities between the two, so the two may be overlapping diseases. However, there are relatively few overlapping cases of SLE and PBC, with a reported prevalence ranging from 0.5 to 3.7% [5].

The independent occurrence of SLE or PBC was found to be related to genetics. For example, based on genome-wide association studies, 38 new SLE-related loci and incompletely shared genetic structures were identified in a large sample population in China [6]; 50 significant loci were identified across the entire genome of 10,516 PBC patients and 20,772 healthy individuals in 6 countries, and potential gene targeting pathways for new loci were identified [7]. However, the occurrence of overlapping cases of SLE and PBC has not yet been fully demonstrated as an accidental event or having a common genetic basis [8].

On this basis, from the perspective of clinical characteristics, a total of 34 overlapping cases of SLE and PBC publicly published before 2015 were analyzed. It was found that they were still more common in women, mostly in the middle-aged and elderly. PBC often occurred before SLE, and some cases were combined with multiple immune diseases (such as Sjogren’s syndrome and hepatocellular carcinoma) [8]. Unfortunately, there is still a lack of research based on genetics to verify the causal relationship between overlapping cases of SLE and PBC.

Mendelian randomization study (MR) is defined as using single nucleotide polymorphisms (SNPs) as instrumental variables to analyze the causal relationship between exposure factors and disease outcomes [9]. Based on a large sample genome-wide association study (GWAS) database, suitable SNPs were screened to effectively reduce the bias of confounding factors on disease outcomes, which were widely used in exploring the causal relationship between phenotypes and diseases. For example, two-sample MR studies evaluated the relationship between SLE and celiac disease and found that SLE could significantly increase the risk of celiac disease [10]; In the European population, inflammatory bowel disease predicted based on 99 SNPs increases the risk of PBC [11]. These and other studies validate the causal relationship between immune diseases at the genetic level, unlike clinical observational studies, which reduce the impact of many confounding factors. Transcriptomics analysis of overlapping diseases, further analyzing the hub genes most likely involved in the development of SLE and PBC overlap, and using hub genes to identify and design drugs that can be used to treat SLE and PBC. For example, the overlapping genes of SLE and venous thromboembolism were identified and found to be closely related to the immune microenvironment, which providing clues for further exploration of the common mechanisms and interactions between the two diseases [12].

Therefore, in this study, firstly, the systematic evaluation of overlapping cases of SLE and PBC would be re-investigated to evaluate the prevalence of overlapping cases. Subsequently, based on genetic evidence from bidirectional dual samples, MR analysis revealed a causal relationship between SLE and PBC. Then, in the analysis of transcriptome data from two samples, potential overlapping genes between SLE and PBC were identified, and optimal models for identifying SLE were constructed based on 11 machine learning algorithms. Finally, based on MR analysis of the causal relationship between overlapping hub genes and SLE/PBC, the robustness of hub genes was verified again. These research contents will provide a deep understanding of the potential common pathophysiological processes of SLE with PBC, and provide a theoretical basis for the implementation of precise diagnosis and treatment in the future.

## Methods

### Literature retrieval of SLE with PBC

This study was searched in the PubMed database using the following search term combinations: [systemic lupus erythematosus or SLE] and [primary biliary cirrhosis or primary cholangitis or PBC] to extract information in the abstract or full text: author, year, and cases.

### Data retrieval

IEU OPEN GWAS database (https://gwas.mrcieu.ac.uk/) was used to retrieve exposure and outcome data. SLE data were obtained from the GWAS dataset (GWAS ID: ebi-a-GCST003156), which dataset was analyzed and constructed by Bentham J et al., consisting of 14,267 Europeans (5201 cases and 9066 controls) with 7,071,163 SNPs [13]. PBC data also were obtained from the GWAS dataset (GWAS ID: ebi-a-GCST005581), which dataset was analyzed and constructed by Liu JZ et al., consisting of 11,375 Europeans (2861 cases and 8514 controls) with 119,756 SNPs [14].

### Genetic instrumental analysis

In order to ensure complete random independence of instrumental variable estimation and exclude the impact of linkage disequilibrium (LD) on outcome factors, a threshold (*P* < 5e − 8) was set to screen SNPs closely related to exposure factors in a genome-wide sense. Next, when the LD parameter was set (*r*^2^ = 0.001 and kb = 10,000), the above SNP data would be filtered for preprocessing to maintain consistency between the exposure effect allele and the outcome effect magnitude. In addition, the F-statistic was calculated to estimate overlapping effects and weak instrumental bias, and *F* < 10 was considered a suspicious bias and excluded.

### MR analysis

Based on the TwoSampleMR software package, the inverse variance weighted (IVW) method was used as the main analysis to evaluate the causal relationship between SLE and PBC. In addition, MR Egger regression, weighted median method, simple estimation based on mode, and weighted estimation based on mode were used as auxiliary analysis methods. Mendelian randomization pleiotropy residual sum and outlier (MR-PRESSO), left one sensitivity analysis, and Cochran’s *Q*-test were used to extensively evaluate MR analysis results. The MR-PRESSO test was used to examine the potential skewness of horizontal pleiotropic by identifying and excluding pleiotropic SNPs with *P* < 0.05 to reassess causal relationships. The leave-one-out method was used to determine the robustness of MR results by excluding SNPs. Cochran’s *Q*-test determined the heterogeneity leading to MR results based on IVW and MR Egger estimates [15].

### Transcriptome in causal relationship validation between SLE and PBC

The microarray expression and clinical data of GSE65391 (924 SLEs and 72 controls) and GSE93170 (6 PBCs and 6 controls) were downloaded from the Gene Expression Omnibus (GEO) database. Based on the limma software package, overlapping differential genes (DEGs) of SLE and PBC were identified, with a threshold of log2 FC > 0 and *P* < 0.05. The biological mechanism of overlapping DEGs based on kyoto encyclopedia of genes and genomes (KEGG) annotations. Based on the STRING database (https://string-db.org), fifteen key hub genes were identified and a PPI network was constructed. On the basis of the 15 bub genes mentioned above, models for identifying SLE were jointly developed based on the least absolute shrinkage and selection operator (LASSO) dimensionality reduction using 11 machine learning algorithms [16]. Further validate the expression of 15 hub genes in SLE patients based on single-cell data downloaded from GEO (GSE163121, GSE162577). The integration and removal of batch effects of diverse materials were processed using the “Harmony” R package. The single-cell analysis process adopts the Seurat method, and the marker genes referred to previous study. The difference analysis between cell clusters was performed using the Kruskal–Wallis H method.

### Statistical analysis

R software (version 4.2.2) for data analysis and plotting. The R software package mainly includes “VariantAnnotation”, “gwasglue”, and “TwoSampleMR”. The estimated value of the causal correlation effect was represented by the odds ratio (OR) and 95% confidence interval (CI) of the risk change of the output factor responding to each increase in standard deviation (SD) of the exposure factor, and the test level was α = 0.05. The classification performance of the models was evaluated based on sensitivity, specificity, accuracy, receiver operating characteristic curve (ROC), and area under ROC curve (AUC).

## Results

### Literature retrieval of SLE with PBC

This study included a total of 9 studies and extracted 125 SLE with PBC (Table 1), including 3 from USA (2.4%), 28 from Turkey (22.4%), 37 from Japan (29.6%), 8 from Italy (6.4%), and 49 from China (39.2%) [5, 8, 17–23].

**Table 1:** SLE with PBC cases derived from the literature

Author	Country	Year	SLE with PBC (*n*)
Chowdhary VR et al [17]	USA	2008	3
Efe C et al [18]	Turkey	2011	2
Takahashi A et al [19]	Japan	2013	3
Shizuma T et al [8]	Japan	2015	34
Floreani A et al [20]	Italy	2015	8
Fan X et al [5]	China	2020	26
Cheng C et al [21]	China	2021	21
Liu Y et al [22]	China	2021	2
Efe C et al [23]	Turkey	2021	26

### Genetic liability to PBC with SLE

**Fig. 1:**
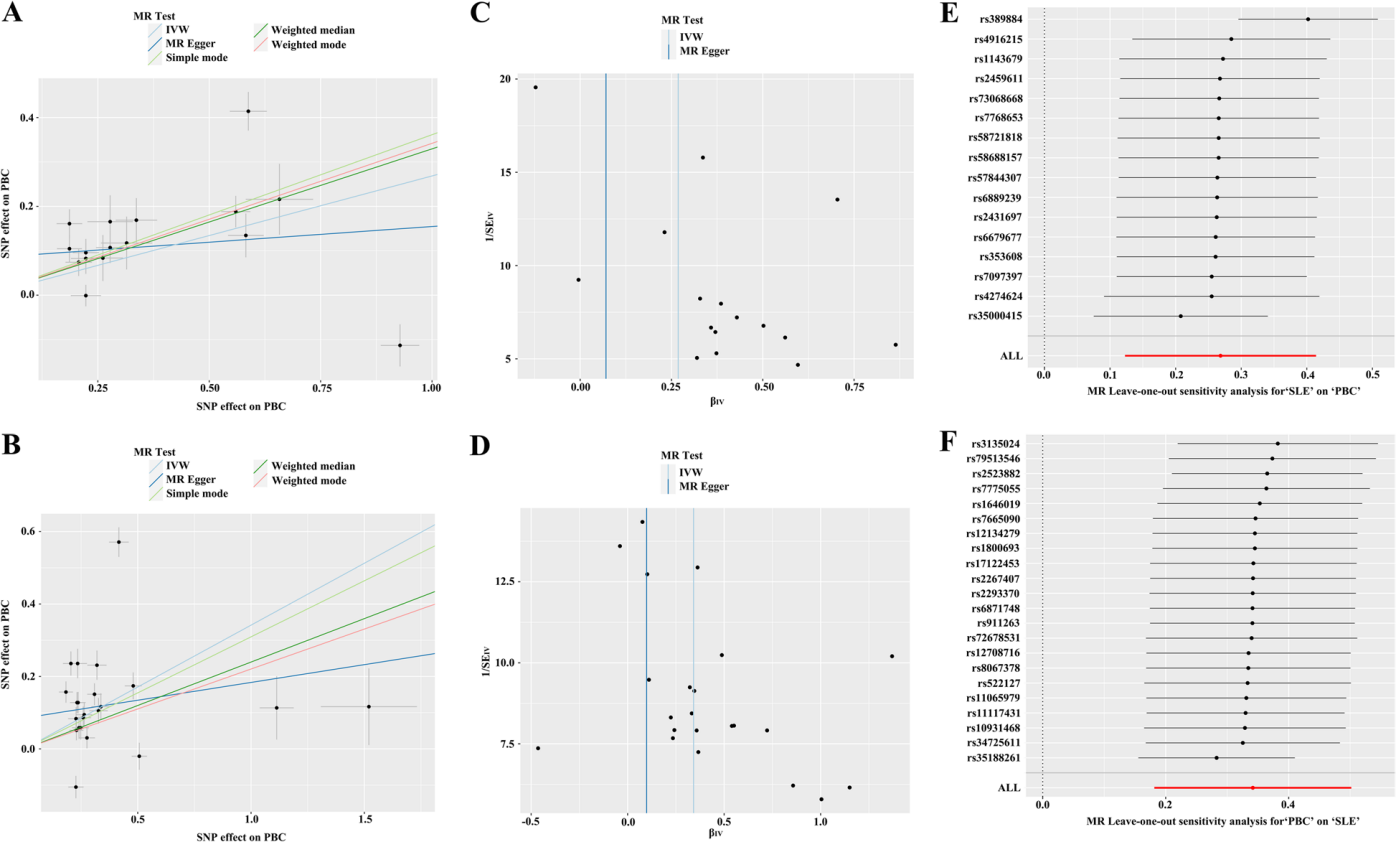
MR estimation of genetic reliability between SLE and PBC. (**A-B**) Scatter plots. The estimation effect of MR methods with slope. Bilateral MR analysis showed that both IVW methods were high-risk factors for SLE or PBC. (**C-D**) Funnel plots. Cochran Q-test showed heterogeneity in the results of bidirectional MR analysis. (**E-F**) Leave-one-out plots. The black dots were used by the IVW method to evaluate causal effects and excluded individual analyses one by one. The red dot indicates the use of IVW for all SNPs. Bidirectional sensitivity testing verified the robustness of MR analysis

After genetic instrumental analysis, 44 SNPs of SLE were used for MR analysis. Two pleiotropic SNPs (rs35000415 and rs389884) were identified through MR-PRESSO test to evaluate horizontal pleiotropy. After removing outliers from the SNPs, only 16 SNPs were found to match the SNPs for PBC. A significant causal relationship was observed between SLE and PBC in the IVW main analysis (OR 1.308, 95% CI, [1.131, 1.513], *P* < 0.001). In addition, the weighted median (OR 1.390, 95% CI, [1.265, 1.523], *P* < 0.001), simple estimation based on mode (OR 1.435, 95% CI [1.231, 1.673], *P* < 0.001), and weighted estimation (OR 1.408, 95% CI [1.218, 1.626], *P* < 0.001) also showed the same results as IVW (Table 2, Fig. 1A). Cochran’s Q-test showed significant statistical significance (IVW, *P* < 0.001, MR Egger, *P* < 0.001), indicating heterogeneity in MR analysis results (Fig. 1C). The leave-one-out method was conducted to verify the robustness of the results found (*P* = 0.124) (Fig. 1E).

**Table 2:** MR estimation of genetic reliability between SLE and PBC

Exposure	Outcome	MR methods	SNPs (*n*)	OR	95% CI	*P*-value
SLE	PBC	MR Egger	16	1.073	0.816–1.412	0.6212
		Weighted median	16	1.390	1.265–1.528	7.4353E − 12
		Inverse variance weighted	16	1.308	1.131–1.513	0.0003
		Simple mode	16	1.435	1.231–1.673	0.0003
		Weighted mode	16	1.408	1.218–1.626	0.0003
PBC	SLE	MR Egger	22	1.103	0.763–1.596	0.6079
		Weighted median	22	1.271	1.161–1.392	2.3155E − 07
		Inverse variance weighted	22	1.407	1.199–1.652	2.9132E − 05
		Simple mode	22	1.363	1.174–1.582	0.0006
		Weighted mode	22	1.247	1.101–1.412	0.0022

### Genetic liability to SLE with PBC

After genetic instrumental analysis, 22 SNPs of PBC were used for MR analysis. Seven pleiotropic SNPs (rs11117431, rs2523882, rs3135024, rs34725611, rs35188261, rs7775055 and rs79513546) were identified through MR-PRESSO test to evaluate horizontal pleiotropy. After removing outliers from the SNPs, 22 SNPs were found to match the SNPs for SLE. A significant causal relationship was observed between SLE and PBC in the IVW main analysis (OR 1.407, 95% CI [1.199, 1.652], *P* < 0.001). In addition, the weighted median (OR 1.271, 95% CI [1.161, 1.392], *P* < 0.001), simple estimation based on mode (OR 1.363, 95% CI [1.174, 1.582], *P* = 0.001), and weighted estimation (OR 1.247, 95% CI [1.101, 1.412], *P* = 0.002) also showed the same results as IVW (Table 2, Fig. 1B). Cochran’s Q-test showed significant statistical significance (IVW, *P* < 0.001, MR Egger, *P* < 0.001), indicating heterogeneity in MR analysis results (Fig. 1D). The leave-one-out method was conducted to verify the robustness of the results found (*P* = 0.169) (Fig. 1F).

### Transcriptome in causal relationship validation between SLE and PBC

**Fig. 2:**
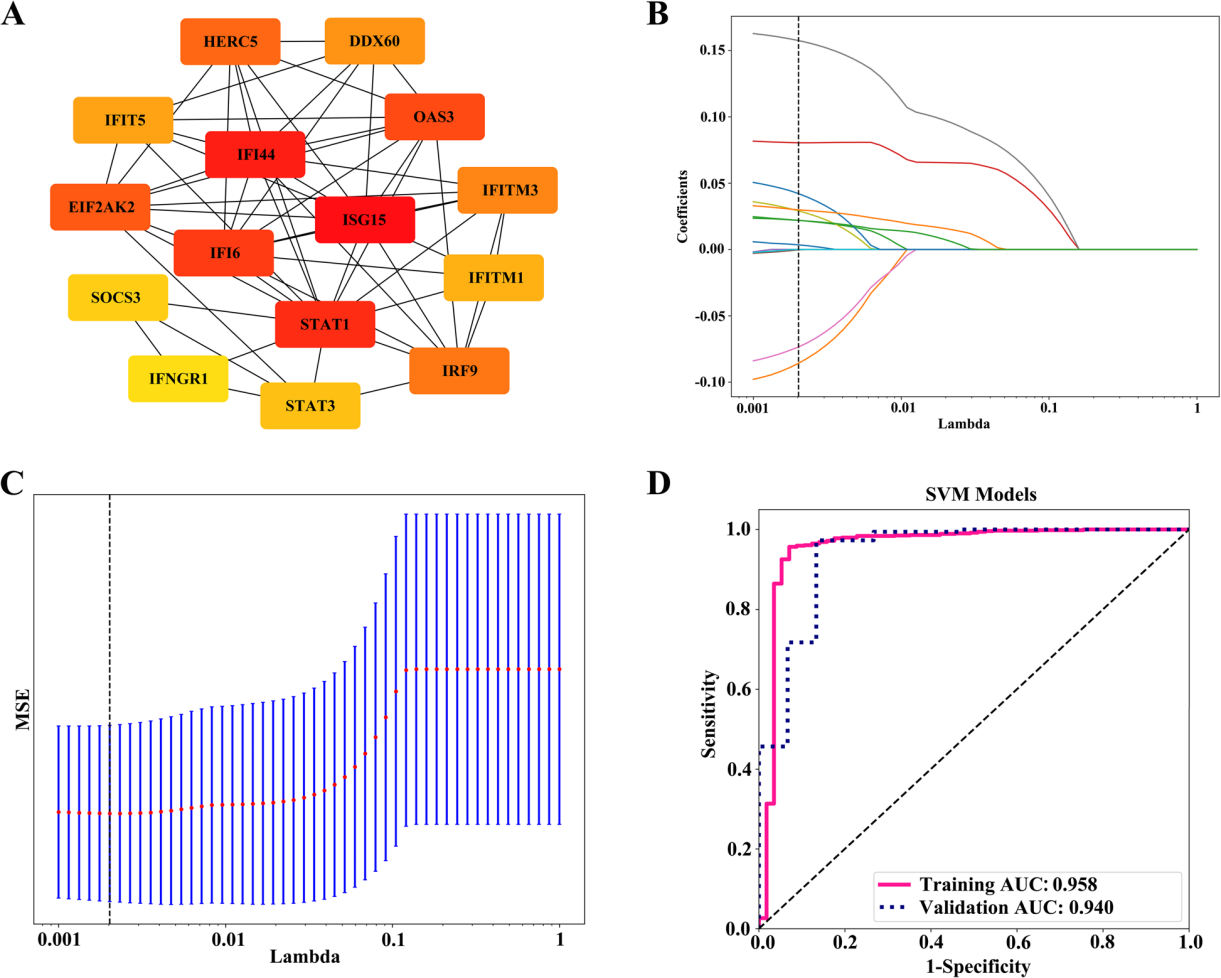
Transcriptome in causal relationship validation between SLE and PBC. **A** Protein–protein interaction network of the top 15 hub genes. **B**–**C** LASSO regression was used for machine learning gene set selection. Coefficient profile diagram (**B**) and cross-validation diagram (**C**). **D** ROC curve of SVM models

**Fig. 3:**
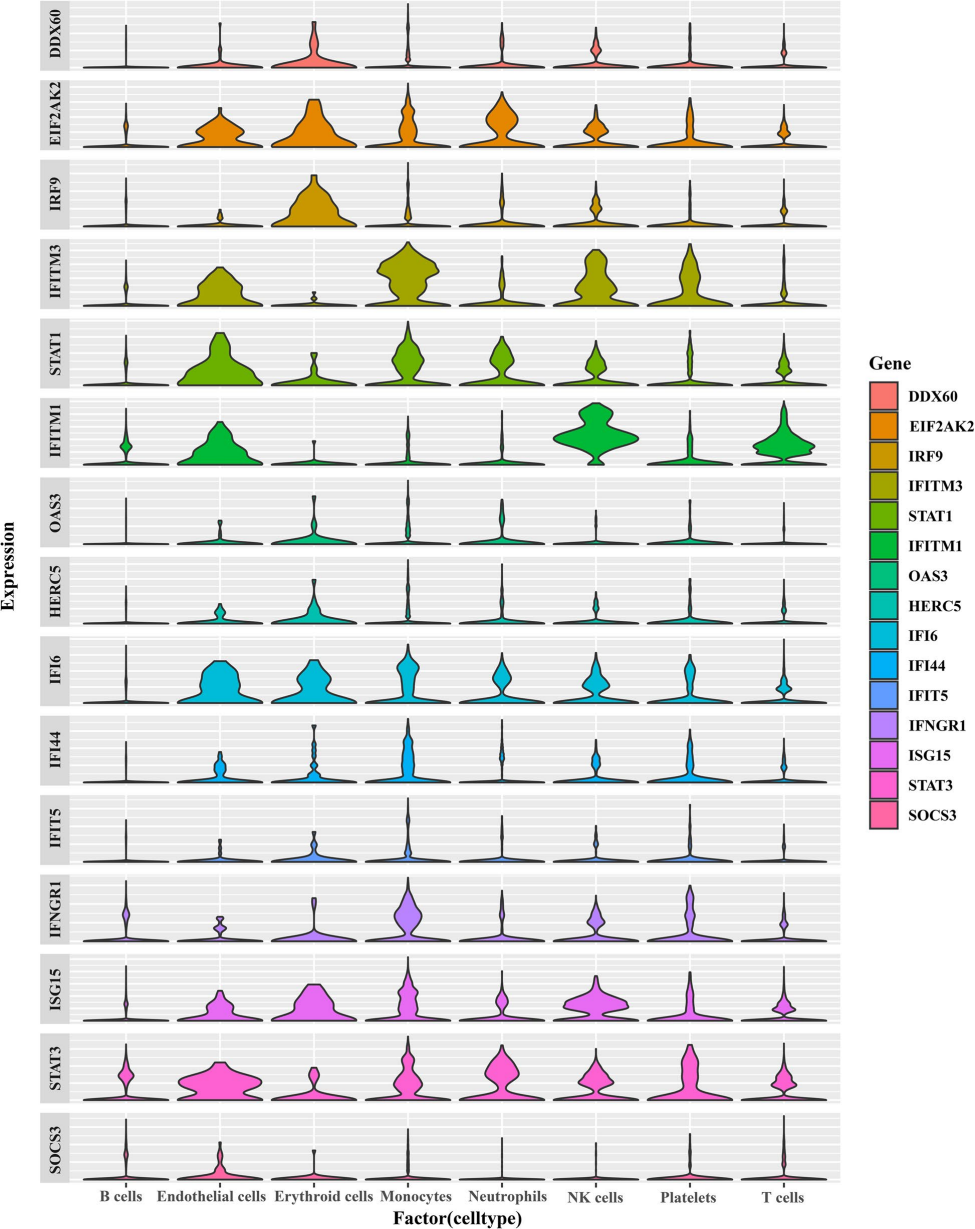
Expression analysis of 15 hub genes in single-cell data. Through single-cell data analysis, among the 15 hub genes, EIF2AK2, IFITM3, STAT1, IFITM1, IFI6, IFI44, IFNGR1, ISG15, STAT3 were highly expressed in various cells (endothecal cells, erythroid cells, monocytes, NK cells, platelets)

In the differential analysis of SLE (GSE65391) and PBC (GSE93170) data, 418 overlapping DEGs were identified. The potential functions of overlapping DEGs had been explored by KEGG, mainly involving the NOD-like receiver signaling pathway, FoxO signaling pathway, cell cycle, TNF signaling pathway, and JAK-STAT signaling pathway. On the basis of the 418 DEGs mentioned above, the PPI network was constructed and imported into the Cytoscape’s Cytohubba plugin to calculate the top 15 hub genes (DDX60, EIF2AK2, IRF9, IFITM3, STAT1, IFITM1, OAS3, HERC5, IFI6, IFI44, IFIT5, IFNGR1, ISG15, STAT3, SOCS3, Fig. 2A). The GSE65391 dataset was divided into a training cohort (*n* = 498) and a validation cohort (*n* = 498) in a ratio of 5 to 5. After LASSO further selection of hub genes, 9 genes (DDX60, EIF2AK2, IRF9, IFITM3, STAT1, IFI6, IFNGR1, ISG15, SOCS3, Fig. 2B–C) were used to construct models for identifying SLE through 11 machine learning algorithms. The results of the training cohort and validation cohort showed high discriminative performance (AUC > 0.90, accuracy > 0.87, sensitivity > 0.87, specificity > 0.87, Table 3). Through comprehensive analysis, it was found that the SVM machine learning algorithm was superior in terms of fitting degree and evaluation reliability. The AUC, accuracy, sensitivity, and specificity of the training and validation queues were 0.96 and 0.94, 0.96 and 0.97, 0.96 and 0.97, 0.93 and 0.87, respectively (Fig. 2D). Surprisingly, through single-cell data analysis, among the 15 hub genes, EIF2AK2, IFITM3, STAT1, IFITM1, IFI6, IFI44, IFNGR1, ISG15, and STAT3 were highly expressed in various cells (Endothecal cells, Erythroid cells, Monocytes, NK cells, Platelets). Unfortunately, hub genes were not significantly expressed in B cells, with some being highly expressed in T cells (Fig. 3).

**Table 3:** Models for identifying SLE patients using 9 hub genes based on 11 machine learning algorithms

Models	AUC	Accuracy	Sensitivity	Specificity	Recall	F1	Cohorts
LR	0.927	0.906	0.907	0.895	0.907	0.947	T
LR	0.966	0.910	0.908	0.933	0.908	0.949	V
NaiveBayes	0.921	0.870	0.868	0.911	0.868	0.925	T
NaiveBayes	0.966	0.879	0.870	1.000	0.870	0.931	V
SVM	0.958	0.955	0.957	0.930	0.957	0.975	T
SVM	0.940	0.965	0.973	0.867	0.973	0.981	V
KNN	0.982	0.891	0.882	1.000	0.882	0.938	T
KNN	0.976	0.879	0.870	1.000	0.870	0.930	V
RandomForest	1.000	0.995	0.995	1.000	0.995	0.997	T
RandomForest	0.938	0.940	0.940	1.000	0.940	0.966	V
ExtraTrees	1.000	1.000	1.000	1.000	1.000	1.000	T
ExtraTrees	0.947	0.950	0.951	1.000	0.951	0.972	V
XGBoost	0.996	0.995	0.996	0.982	0.996	0.997	T
XGBoost	0.964	0.920	0.918	0.933	0.918	0.955	V
LightGBM	0.993	0.967	0.968	0.965	0.968	0.982	T
LightGBM	0.967	0.925	0.924	0.933	0.924	0.958	V
GradientBoosting	0.979	0.951	0.951	0.947	0.951	0.973	T
GradientBoosting	0.934	0.915	0.913	1.000	0.913	0.952	V
AdaBoost	0.972	0.905	0.900	0.965	0.900	0.946	T
AdaBoost	0.974	0.930	0.924	1.000	0.924	0.960	V
MLP	0.938	0.897	0.895	0.930	0.895	0.942	T
MLP	0.961	0.925	0.924	0.933	0.924	0.958	V

### SOCS3 genetic reliability for SLE and PBC

Due to the low significance of SNPs (*P* < 5e − 8) in the SOCS3 (GWAS ID: prot-a-2797) dataset, the new threshold was set to *P* < 5e − 5. In the SOCS3’s genetic reliability for SLE, Cochran’s *Q*-test showed no significant statistical significance (IVW, *P* = 0.513, MR Egger, *P* = 0.536), indicating no heterogeneity in MR analysis results. In the IVW main analysis, there was no significant causal relationship between SOCS3 and SLE (OR 0.992, 95% CI [0.936, 1.051], *P* = 0.787), while other methods showed the same results. The leave-one-out method verified the robustness of the results (*P* = 0.207). In the SOCS3’s genetic reliability for PBC, Cochran’s *Q*-test did not show significant statistical significance (IVW, *P* = 0.916, MR Egger, *P* = 0.906), indicating no heterogeneity in MR analysis results. In the IVW main analysis, there was a significant causal relationship between SLE and PBC (OR 1.387, 95% CI [1.049, 1.835], *P* = 0.022, Table 4), while other methods showed the same results without statistical significance. The leave-one-out method verified the robustness of the results (*P* = 0.632).

**Table 4:** MR estimation of genetic reliability between SOCS3 and SLE/PBC

Exposure	Outcome	MR methods	SNPs (*n*)	OR	95% CI	*P*-value
SOCS3	SLE	MR Egger	62	0.937	0.843–1.041	0.2289
		Weighted median	62	0.975	0.883–1.075	0.6062
		Inverse variance weighted	62	0.992	0.936–1.051	0.7866
		Simple mode	62	0.907	0.741–1.111	0.3505
		Weighted mode	62	0.963	0.870–1.066	0.4701
SOCS3	PBC	MR Egger	4	1.761	0.731–4.242	0.3346
		Weighted median	4	1.345	0.962–1.882	0.0829
		Inverse variance weighted	4	1.387	1.049–1.835	0.0218
		Simple mode	4	1.282	0.844–1.948	0.3286
		Weighted mode	4	1.291	0.798–2.090	0.3741

## Discussion

At present, the clinical characteristics and pathophysiology of SLE with PBC overlap cases are not clear. Further analysis is needed to determine whether the overlap between the two will affect the risk of SLE or PBC occurrence and development, as well as whether it interferes with treatment effectiveness. This study investigated the epidemiological characteristics of SLE with PBC, which was similar to literature reports and had a low incidence rate. In addition, the potential causal relationship between SLE with PBC was elucidated from a genetic perspective. The MR analysis results showed that both SLE and PBC were high-risk factors for the occurrence and development of the other party. In addition, hub genes were identified through transcriptome overlap analysis, and their roles in SLE were validated through machine learning algorithms. Finally, based on MR analysis, the causal relationship between hub gene SOCS3 and SLE/PBC was verified. This study provides a new perspective for the precise diagnosis and treatment of SLE with PBC overlapping cases and also provides a theoretical basis for future research on common potential mechanisms and potential therapeutic targets.

Numerous well-known, the prevalence of SLE with PBC is low. According to statistics, a total of 125 cases had been reported, most of which were case reports, with one-third of the cases coming from China, and 75 cases had been reported in the past three years. The clinical characteristics were still the same as previous findings, and were more common in middle-aged and elderly women, with diverse clinical manifestations. However, observational studies had shown that SLE with PBC may have potential independent clinical features compared to PBC, such as anti-mitochondrial antibodies, hemoglobin, and albumin. In addition, SLE with PBC was a high-risk factor for adverse events related to liver cirrhosis and liver cancer. For example, univariate analysis showed that SLE with PBC was associated with the development of HCC (hazard ratio = 5.02) [23]. Unfortunately, no significant difference in survival was found between SLE with PBC and PBC alone [5]. However, the application of observational studies on confounding factors of diversity and reverse causal effects in causal inference. To reduce confounding factors, observational studies can typically use propensity scoring methods to match baseline characteristics of exposed and unexposed patients, resulting in effect estimates similar to those of randomized controlled trials [24]. Unfortunately, matching queues during propensity score analysis results in a large amount of data loss, which may weaken the reliability of the conclusion [25]. Therefore, more new technologies are still needed to explore the causal relationship between SLE and PBC.

MR is a genetic variable analysis that follows mendelian laws of inheritance, allowing SNPs to infer the causal relationship between exposure factors and clinical outcomes. Due to the random segregation of alleles, MR can reduce bias caused by confounding factors. In addition, since genetic variation occurs before the disease, the order of the two cannot be reversed, MR can also avoid interference from reverse causal relationships [26]. GWAS is a database of MR analysis sources that can be used to indicate that SNPs present at multiple locations in the genome can be associated with specific phenotypes [27]. In this study, the genetic causal relationship between SLE and PBC was elucidated in a dual sample bidirectional manner based on large sample whole genome association analysis. In this study, there were 15674 significant loci in SLE and 1135 significant loci in PBC. However, after linkage disequilibrium analysis, there were 44 remaining SNPs in SLE and 22 remaining SNPs in PBC, and these SNPs did not overlap. The use of IVW as the main analysis method not only indicated a causal relationship between the increased risk of PBC and SLE (OR 1.308, 95% CI [1.131, 1.513], *P* < 0.001), but also indicated a causal relationship between the increased risk of SLE and PBC (OR 1.407, 95% CI [1.199, 1.652], *P* < 0.001). In addition, the three methods of weighted media, simple estimation based on mode, and weighted estimation also supported the analysis results of IVW. However, the results of bidirectional MR analysis showed significant heterogeneity, which may be related to the number of SNPs detected. Although the sample size of SLE and PBC was similar and both were European populations, there was a significant difference in the number of SNPs detected between the two (SLE, 7071163; PBC, 119756), which led to a large number of SNPs in SLE whose genetic value had not been elucidated. Excitedly, sensitivity testing supported the stability and accuracy of causal results between SLE and PBC.

Transcriptome overlap analysis is a genetic validation method for analyzing the relationship between two diseases. For example, based on three datasets of polycystic ovary syndrome and recurrent implantation failure, 12 disease overlapping genes were identified using weighted gene co expression networks, functional enrichment analysis, and three machine learning algorithms, which potential pathogenic associations and mechanisms between the two were explored [28]; The inflammatory immune pathway of calcified aortic valve disease (CAVD) related to chronic kidney disease (CKD) had been revealed, providing new insights for future serum based diagnosis and treatment interventions for CKD combined with CAVD [29]. In this study, 418 upregulated overlapping genes were identified using the GEO dataset of SLE and PBC, and their biological functions were found to be related to TNF signaling pathway and JAK-STAT signaling pathway, which was similar to previous reports [7]. Subsequently, based on the PPI network, the top 15 most valuable hub genes were identified. Through single-cell data analysis, multiple hub genes had also been validated to exhibit high expression in various cells. After further screening by LASSO, 9 genes (DDX60, EIF2AK2, IRF9, IFITM3, STAT1, IFI6, IFNGR1, ISG15, SOCS3) were selected and presented to 11 machine learning algorithms to validate their value for SLE. The results showed that the machine learning models constructed by these 9 genes were effective in distinguishing SLE patients from non-SLE patients, with high efficiency in AUC, accuracy, sensitivity, and specificity. In addition, among the 9 genes mentioned above, 6 genes (DDX60, EIF2AK2, IRF9, IFI6, IFNGR1, ISG15) have been elucidated to be associated with SLE, but no reports have been found to be related to PBC; Both STAT1 and SOCS3 were found to be associated with SLE and PBC; IFITM3 has not been reported to be associated with SLE or PBC. SOCS3 could be lost by the IL10R1 gene allele, inducing genetic susceptibility to SLE disease in Caucasian populations [30]; The expression of SOCS3 mRNA was more inhibited in the PBC liver, but it was unlikely to explain its loss of Stat3 DNA binding in PBC [31]. The causal relationship between the SOCS3 gene and SLE and PBC will be further confirmed based on MR analysis. The results showed that elevated levels of the SOCS3 gene were a high-risk factor for increased risk of PBC (OR 1.387, 95% CI [1.049, 1.835], *P* = 0.022), and there was no causal relationship with SLE (OR 0.992, 95% CI [0.936, 1.051], *P* = 0.787).

This study had some advantages and limitations. The advantages of this study: Firstly, MR analysis could evaluate the bidirectional causal relationship between SLE and PBC in both directions and reduce confounding factors. Then, a novel method based on MR analysis was used to explore the clinical prognostic characteristics of SLE with PBC overlapping cases, which could to some extent overcome the limitation of insufficient case samples. Finally, unlike other MR studies, this study used transcriptome overlap analysis to re-validate the potential common mechanism between the two. Limitations of this study: Firstly, both population groups were European, and although the sample size was similar, there was a significant difference in the number of SNPs tested. Subsequently, the representativeness of SNPs’ significance was insufficient, and cross-validation of SNPs’ significance was still needed in more samples. Then, MR analysis exhibits heterogeneity, which may be related to significant differences in SNPs between the two samples and insufficient utilization of SNPs information. Thirdly, similar to other studies, based on the summary data analysis used by GWAS, individual data could not be obtained and other confounding factors could not be excluded. Fourthly, the microarray expression sequencing platforms for SLE and PBC downloaded from GEO were different, and there was a significant difference in sample size, which was also an important limitation. Finally, sensitivity analysis could not completely eliminate potential level pleiotropy [32, 33].

## Conclusions

This study is the first attempt to use MR analysis to explore the bidirectional causal relationship between SLE and PBC, which can to some extent overcome the limitation of insufficient overlapping case samples in SLE with PBC. The results showed that both SLE and PBC were associated with an increased risk of development in the other party. The analysis of transcriptome overlap hub genes provides a new perspective for the precise diagnosis and treatment of SLE with PBC overlap cases and also provides a theoretical basis for future research on common potential mechanisms and potential therapeutic targets.

## Data Availability

The datasets used and/or analyzed during the current study are available from the corresponding author on reasonable request.

## Abbreviations

SLE: Systemic lupus erythematosus
PBC: Primary biliary cirrhosis
MR: Mendelian randomization
SNPs: Single nucleotide polymorphisms
GWAS: Genome-wide association study
IVW: Inverse variance weighted
MR-PRESSO: Mendelian randomization pleiotropy residual sum and outlier
GEO: Gene expression omnibus
DEG: Overlapping differential gene
GO: Gene ontology
KEGG: Kyoto encyclopedia of genes and genomes
LASSO: Least absolute shrinkage and selection operator
OR: Odds ratio
SD: Standard deviation
ROC: Receiver operating characteristic curve
AUC: Area under curve
CI: Confidence interval
CVAD: Calcified aortic valve disease
CKD: Chronic kidney disease
LD: Linkage disequilibrium
